# MapReduce-based big data classification model using feature subset selection and hyperparameter tuned deep belief network

**DOI:** 10.1038/s41598-021-03019-y

**Published:** 2021-12-17

**Authors:** Surendran Rajendran, Osamah Ibrahim Khalaf, Youseef Alotaibi, Saleh Alghamdi

**Affiliations:** 1 Center for Artificial Intelligence and Research (CAIR), Chennai Institute of Technology, Chennai, India; 2 Al-Nahrain Nanorenewable Energy Research Center, Al-Nahrain University, Baghdad, Iraq; 3 Department of Computer Science, College of Computer and Information Systems, Umm Al-Qura University, Makkah, Saudi Arabia; 4 Department of Information Technology, College of Computers and Information Technology, Taif University, Taif, Saudi Arabia

**Keywords:** Computer science, Information technology

## Abstract

In recent times, big data classification has become a hot research topic in various domains, such as healthcare, e-commerce, finance, etc. The inclusion of the feature selection process helps to improve the big data classification process and can be done by the use of metaheuristic optimization algorithms. This study focuses on the design of a big data classification model using chaotic pigeon inspired optimization (CPIO)-based feature selection with an optimal deep belief network (DBN) model. The proposed model is executed in the Hadoop MapReduce environment to manage big data. Initially, the CPIO algorithm is applied to select a useful subset of features. In addition, the Harris hawks optimization (HHO)-based DBN model is derived as a classifier to allocate appropriate class labels. The design of the HHO algorithm to tune the hyperparameters of the DBN model assists in boosting the classification performance. To examine the superiority of the presented technique, a series of simulations were performed, and the results were inspected under various dimensions. The resultant values highlighted the supremacy of the presented technique over the recent techniques.

## Introduction

Big data is a group of information that cannot be processed, captured, and managed by means of traditional software implements in a particular interval. This can be a higher growth rate, huge and differentiated data resource^1^. It needs novel processing modes to have strong decision-making control, understanding, optimization capability, and discovery powers. Big data analysis is performed by many approaches, such as databases, web crawlers, analysis, data warehouses, big data mining, and visualization algorithms. Among others, web crawlers are a widespread analysis technology^2^. They could extract text data/numerical values from a webpage and set up it to analyze information. In addition to Python language, this technique could be executed with few software packages. The big data mining algorithm needs to manage a huge amount of information. Furthermore, the speediness of handling is deliberate. This algorithm includes optimization technologies (such as GDA and PSO algorithms), scientific modeling or other predictive technologies (such as NN, NB, roughset, DT), decision analysis technologies (such as multicriteria decision, gray decision, and so on), performance assessment technologies (for example, fuzzy comprehensive evaluation and data envelopment analyses). Since this mining information was hardly examined and big data in the historical, particularly used to engineer problems, it has is a very big problem while employing this mining information algorithm to big data^3^.

Clustering and classification are the two key classes of algorithms in mining information^4^. Nevertheless, the performances of classification and clustering methods are considerably caused by the increasing dataset dimension because the algorithm in this category operates on the dataset dimension. Additionally, the drawback of higher dimension datasets includes redundant data, higher module construct time, and degraded quality, which makes information analyses highly complicated. To resolve these problems, the selection of features is employed as a major preprocessing phase for choosing subsets of features from a large dataset and increases the accuracy of clustering and classification models, which triggers foreign, ambiguous, and noisy data elimination^5^. The FS method depends upon search techniques and a performance assessment of subsets. Since the preprocessing stage, feature selection was essential for removing duplications, minimizing the amount of information, and irrelevant and unnecessary characteristics. It has various techniques to select the feature that assists in choosing the actual dataset as the effective feature. Filter, embedded and wrapper are the 3 approaches of the FS model^6^. The selection of features should achieve 2 aims: to eliminate/reduce the amount of FS and increase the output performance. As already mentioned, meta heuristics in previous decades simulate organisms’ collective behaviors. In particular, this algorithm has generated an important development in several regions associated with optimization^7^. The optimal selection is made by a metaheuristic algorithm; in a rational interval, the cloud generates better solutions^8^. Sometimes, it is a better solution to mitigate the limitation of comprehensive time-consuming searches^9^. Various metaheuristic methods, alternatively, suffer from the optimum location, missing search multiplicity and imbalance among exploitative and explosive performances^10^. Recently, EA has shown itself to be efficient and attractive for solving challenges using optimizations. There are few approaches, such as PSO, CSA^11^, GA^12^, and ACO algorithms^13^. PSO was hybridized for constant search space issues in the works with another metaheuristics approach.

This study focuses on the design of a big data classification model using chaotic pigeon inspired optimization (CPIO)-based feature selection with an optimal deep belief network (DBN) model^14^. The proposed model is executed in the Hadoop MapReduce environment to manage big data. Initially, the CPIO algorithm is applied to select a useful subset of features. In addition, the Harris hawks optimization (HHO)-based deep belief network (DBN) model is derived as a classifier to allocate appropriate class labels. The design of the HHO algorithm to tune the hyperparameters of the DBN model assists in boosting the classification performance. To examine the superiority of the presented technique, a series of simulations were performed, and the results were inspected under various dimensions. This paper structure is defined as follows. In “Results” section, the limitation of the proposed research work was identified through a literature review. Hadoop map reduction models defined in “Discussion” section. In “Methods” section, the performance analysis of the proposed system is briefly elaborated. This is followed by conclusions and some probable future directions are recommended in “Conclusion” section. In Al-Thanoon et al., BCSA was stimulated by natural phenomena to perform the FS method. In BCSA, the flight length variable plays a significant part in the performance^15^. To enhance the classification performances by rationally elected features, a development of defining the flight length parameters through the concepts of the opposition-based learning method of BCSA is presented. BenSaid and Alimi proposed an OFS method that solves these problems^16^. The presented method named MOANOFS explores the new developments of the OML method and conflict resolution method (Automated Negotiation). MOANOFS employs a 2-decision level. Initially, deciding k(s) among the learner (OFS method) is trustful (trust value/higher confidence). This selected k learner will take part in the next phase in which this presented MANOFS technique is incorporated.

In Pooja et al., the TC-CMECLPBC method is projected. Initially, the features and data were collected from large climate databases^17^. The TCC model is employed to find the comparison among the features to select appropriate features through high FS precision. The clustering method consists of 2 stages, maximization (M) and expectation (E), for discovering the maximal likelihood of grouping information into clusters. Next, the clustering results are provided to linear program boosting classifiers to improve the predictive performance. Lavanya et al. examine FS techniques such as rough set and entropy on the sensor's information^18^. Additionally, a representative method of FW is presented that represents Twitter and sensor information effectively for additional analyses of information. Few common classifications, such as NB, KNN, SVM, and DT, are employed to validate the efficiency of the FS. An ensemble classification method is presented that is related to many advanced methods. In Sivakkolundu and Kavitha, a new BCTMP-WEABC method is presented to predict upcoming outcomes with high precision and less time consumption^19^. This method includes 2 models, FS and classifier, to handle a large amount of information. Baldomero-Naranjo et al. proposed a strong classification method depending upon the SVM method that concurrently handles FS and outlier discovery^20^. The classifiers are made to consider the ramp loss margin error and involve budget constraints for limiting the number of FSs. The search of classifiers is modeled by a mixed integer design through a large M parameter. Two distinct methods (heuristic and exact) are presented for solving this method. The heuristic method is authenticated by relating the quality of the solution given to this method using an accurate method.

Guo et al. proposed a WRPCSP method for executing the FS method. Next, the study incorporates BN and CBR systems for reasoning knowledge^21^. According to the possible reasoning and calculation, WRPCSP algorithms and BN permit the presented CBR scheme to work in big data. Furthermore, to solve these problems created with a large number of features, this study also proposed a GO method for assigning the computation process of big data for similar data processing. Wang et al. proposed a big data analytics approach to the FS process for obtaining each explanatory factor of CT, which will shed light on the fluctuations of CT^22^. Initially, the relative analyses are executed among all 2 candidate factors through mutational data metrics to construct the experiential system. Next, the system deconvolutions are explored to infer the direct dependencies among the candidate’s factors and the CT by eliminating the effect of transitive relationships from the system. In Singh and Singh, the 4-phase hybrid ensemble FS method was proposed. Initially, the datasets are separated by the cross validation process^23^. Next, several filter approaches that depend on the weight score are ensembles for generating a rating of features, and then the consecutive FS method is used as a wrapper method for obtaining the best subsets of features. Finally, the resultant subsets are treated for the succeeding classifier's task. López et al. proposed a distributed feature weight method that accurately estimates feature significance in a large dataset with the popular method RELIEF in smaller problems^24^. The solution named BELIEF integrates new redundant removal measures that generate schemes related to this entropy but with low time costs. Furthermore, BELIEF provides a smoother scale-up, while additional cases are needed to increase the accuracy of the estimation.

## Results

In this study, a new big data classification model is designed in the MapReduce environment. The proposed model derived a novel CPIO-based FS technique, which extracts a useful subset of features. In addition, the HHO-DBN model receives the chosen features as input and performs the classification process. The detailed working of these processes in the Hadoop MapReduce Environment is offered in the following sections. An essential component that develops the Hadoop architecture is as follows: Hadoop Distributed File System (HDFS): Initially, it can be a Google File System. This component was a distributed file system utilized as distributed storage to data; additionally, it gives access to information with maximum throughput. Hadoop YARN (MRv2): This component has been responsible for job scheduling and managing cluster resources. Hadoop MapReduce: Initially, Google’s MapReduce, these modules are scheme dependent upon YARN to the parallel process of data.

There are several studies compared with Hadoop, namely, Mahout, Hive, Hbase, and Spark. The most essential feature that describes Hadoop is which the HDFS is a maximum fault tolerance to hardware failure. Certainly, it can be capable of repeatedly handling and resolving these cases. Additionally, HDFS can interface among the nodes going to cluster to manage the data, i.e., to rebalance them^25^. The model of data storing the HDFS was carried out using the MapReduce structure. While Hadoop has expressed mainly in Java and C languages, it can be near several other programming languages. The MapReduce structure allows separation of nodes going to cluster the task, which is also finished. An essential disadvantage of Hadoop is the absence of execution capable of real-time tasks. However, it could not be a vital restriction because of these particular features, another technology is utilized. MapReduce automatically parallelizes and applies the program to a large cluster of commodity technologies. It mechanism by break model as to 2 stages, the map as well as reduce stage. All the stages are key-value pairs as input as well as output, this kind of that can be elected as programmer. The map and reduces operations of MapReduce are combined and determined in terms of data structured form (key, value) pairs. The calculation obtains the group of input key-value pairs and makes the group of output key-value pairs. The map as well as reduce operations in Hadoop MapReduce is the subsequent common procedure:

$$map:({k}_{1},{v}_{1})\to list({k}_{2},{v}_{2})$$

$$reduce:\left({k}_{2}, list\left({v}_{2}\right)\right)\to list\left({v}_{2}\right)$$

### Design of CPIO-FS technique

At this stage, the CPIO-FS technique is applied to derive a subset of features. PIO has been a newly developed bioinspired technique extremely utilized for solving the optimized issue. It can be dependent upon social organisms of swarms that utilize the base of learning. It tried to mathematically enhance the solution quality based on the natural performance of the swarm to adapt to the position and velocity of all individuals. The homing nature of pigeons derives from 2 important functions, mapping and compassing and landmark functions. Map and compass operator: At this point, the rules were explained as the place ${X}_{i}$ and velocity ${V}_{i}$ of pigeon $i$, and the position and velocity in the $D$-dimensional search space were maximized at each iteration. The new place and velocity of pigeons $i$ at the $t$-th iteration have been defined utilizing the provided function in Eqs. (1)–(2):

1 $${V}_{i}\left(t\right)={V}_{i}\left(t-1\right)\cdot {e}^{-Rt}+rand\cdot \left({X}_{g}-{X}_{i}\left(t-1\right)\right)$$

2 $${X}_{i}\left(t\right)={X}_{i}\left(t-1\right)+{V}_{i}\left(t\right)$$

where $R$ stands for the mapping and compassing factors, rand demonstrates the arbitrary value in [0–1], and ${X}_{g}$ illustrates the present global optimum place, which is obtained in the evaluation of every location^26^.

Landmark operator: During this metric, the partial amount of pigeons is diminished from every generation. To accomplish the target immediately, residual pigeon flies to the destination place^27^. Let ${X}_{c}$ be the middle place of pigeons, and the place upgrading rule of pigeon $i$ at the t‐th iteration has been written as Eqs. (3)–(5):

3 $${N}_{p}\left(t\right)=\frac{{N}_{p}\left(t-1\right)}{2}$$

4 $${X}_{c}\left(t\right)=\frac{\sum {X}_{i}\left(t\right)\cdot fitness\left({X}_{i}\left(t\right)\right)}{{N}_{p}\cdot \sum fitness\left({X}_{i}\left(t\right)\right)}$$

5 $${X}_{i}(t)={X}_{i}(t-1)+rand\cdot ({X}_{c}(t)-{X}_{i}(t-1))$$

where ${N}_{p}$ refers to the amount of pigeons, but the fitness is the cost function of pigeons. To reduce optimization, the target function has been elected from the rate of minimum.

### Optimal feature selection process

The FF objective is a terminology utilized to estimate the solution. The FF evaluates the solution, which is a subset of obvious features, by means of the true positive rate (TPR), false positive rate (FPR), and number of features. The number of features comprises FF, and there are features obtainable without affecting TPR or FPR. During these cases, it can be necessary to eliminate individual features. Equation (6) schemes the function executed to estimate the fitness of the pigeon or solution.

6 $$FF={w}_{1}*\frac{SF}{NF}+{w}_{2}*FPR+{w}_{3}*\frac{1}{TPR}$$

where $SF$ determines the number of elected features, $NF$ refers to the entire feature and ${w}_{1}+{w}_{2}+{w}_{3}=1$. The measures of weights are set as ${w}_{1}=0.1$ and ${w}_{2}={w}_{3}=0.45$ because TPR and FPR are important. An initial method of projected PIO-FS defines the outcome or pigeon by vector with length, which is similar to feature count. As the PIO techniques fundamental approach acts on the place of pigeon regularly, the proposed PIO for FS explains the solution inference as vector of a definite length, but the measures of place and velocity vectors are arbitrary values in 0 and 1.

Usually, the velocity of pigeons is monitored by a sigmoidal function that has been utilized to transfer velocity as a binary version by utilizing Eq. (7). To resolve the binarized SI technique, the pigeon place is upgraded depending upon the sigmoid function value and the possibilities of arbitrarily uniform values in 0 and 1 by Eq. (8). The residual manner was operated similar to convention PIO except for the upgrading place of landmark operators. In addition, the sigmoid function was implemented transmission, and the velocity and place were upgraded as:

7 $$S\left({V}_{i}\left(t\right)\right)=\frac{1}{1+{e}^{\frac{-{v}_{i}}{2}}}$$

8 $$X(t{)}_{\left(i,p\right)}[i]=\left\{\begin{array}{l}1, if\left(S\left({V}_{i}\left(t\right)\right)>r\right)\\ 0, otherwise\end{array}\right.$$

where ${V}_{i}(t)$ stands for the pigeon velocities from iteration $t$ and $r$ refer to the uniformly arbitrary values.

## Discussion

The Design of HHO-DBN Model is discussed here. The features are passed into the DBN model to perform the classification process. The DBN was a probability generation approach that is opposite the classic discriminative approach. This network is a DL technique that is stacked by RBM and trained in a greedy approach. The resultant prior layer was utilized as the input of the succeeding layer. Eventually, the DBN network has been generated. In the DBN, hierarchical learning has been simulated as the framework of the human brain. All the layers of the deep network are regarded as a logistic regression (LR) approach. The joint distribution function of $x$ and ${h}^{k}$ in Layer $l$ is in Eq. (9).

9 $$p\left(x,{h}^{1},{h}^{2}, \dots ,{h}^{l}\right)= \left(\prod_{k=0}^{l-2}P\left({h}^{k}|{h}^{k+1}\right)\right)P\left({h}^{l-1},{h}^{l}\right).$$

Input data of the DBN method compose the 2D vector reached in preprocessing. The RBM layers were trained one-by-one in pretrained. The following visible variable is a duplicate hidden variable from the preceding layer^28^. The parameter is transmitted in a layerwise approach, and the features have been learned in the preceding layer. The LR is maximum layers trained by fine‐tuning, where the cost function has been revised using BP for optimizing the weight $w.$ The 2 steps are contained in the procedure of trained a DBN technique. All the RBM layers are unsupervised trained, input has been mapped as to distinct feature space, and data has been saved about feasible. Afterward, the LR layer was added on top of the DBN as supervised classification. Figure 2 demonstrates the framework of the DBN model.

**Figure 1:**
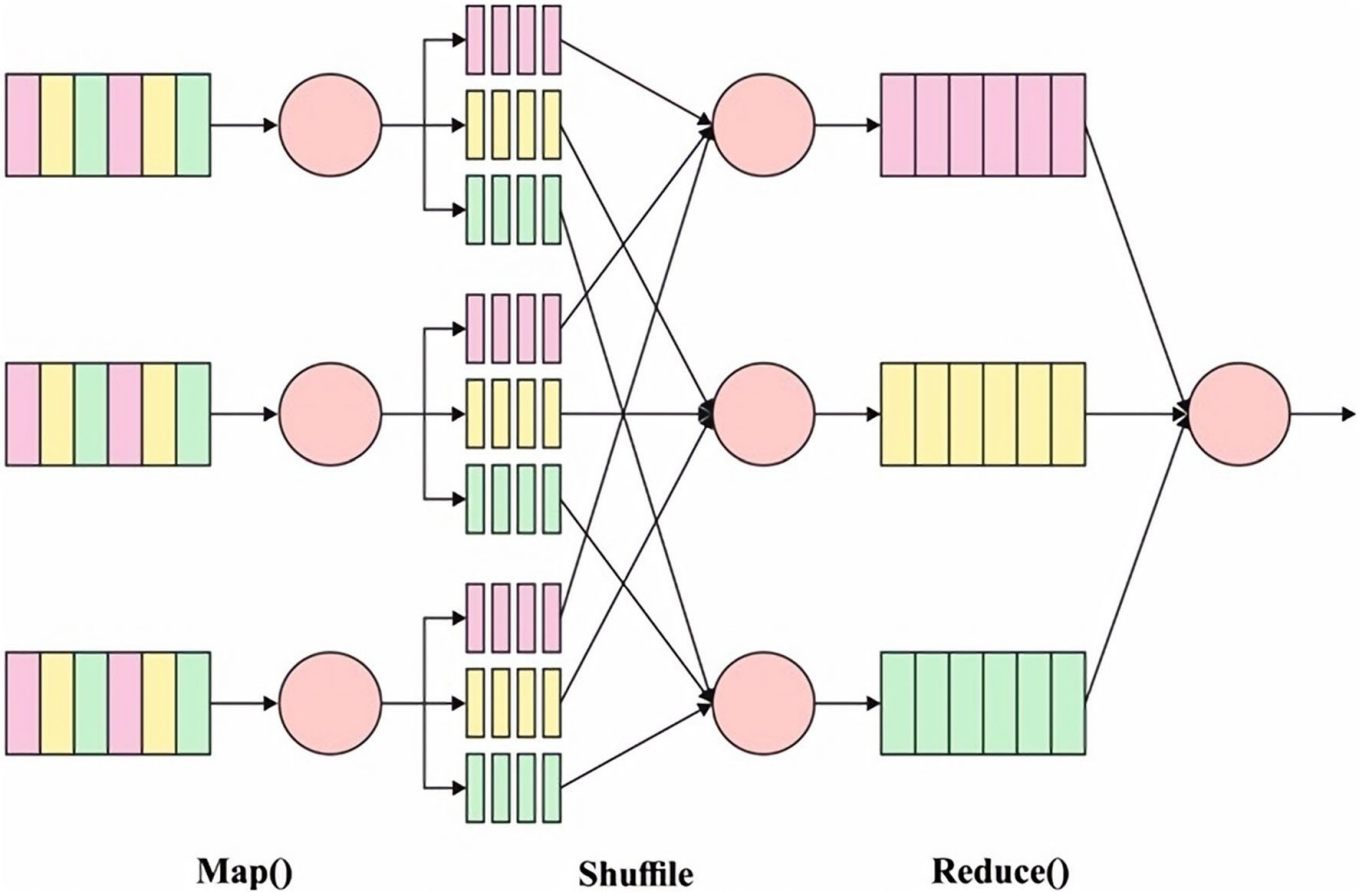
Framework of Mapreduce in Hadoop. It consists of map as well as reduce operations. The map stage obtains the various inputs and spilt and shuffled it. During this reduction stage, all reduce tasks procedure the input in-between data with a decrease function and create output data. Figure illustrates the block diagram of MapReduce.

**Figure 2:**
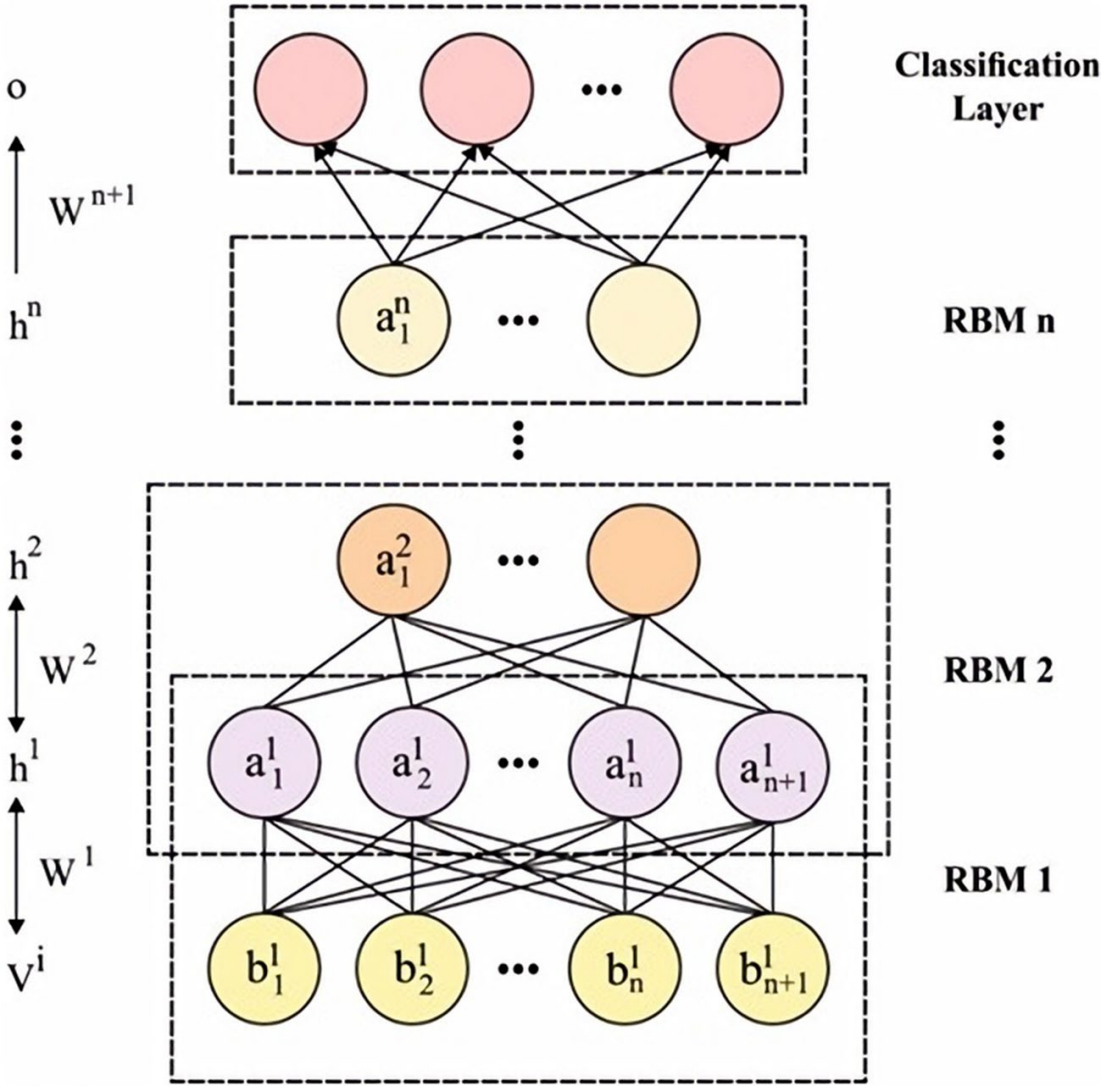
DBN structure. The hyperparameters of the DBN model take place using the HHO algorithm to boost the classifier results. During the HHO, the candidate solution is the Harris hawks, and an optimum/global solution has been planned prey. Therefore, HHO illustrates the exploratory and exploitative phases.

The HHO technique is simulated as the hunting performance of Harris hawk to rabbit. If the rabbits have considerable energy, the Harris hawk explores the definitional domain $[$LB, $UB]$ with the subsequent formula in Eq. (10):

10 $${x}_{i}\left(t+1\right)=\left\{\begin{array}{ll}{x}_{r}-{r}_{1}\left|{x}_{r}-2{r}_{2}{x}_{i}\left(t\right)\right|& q\ge 0.5\\ ({x}_{b}-{x}_{m})-{r}_{3}(LB+{r}_{4}(UB-LB)& q<0.5\end{array}\right.$$

where ${x}_{i}(t+1)$ implies the place of the $i$th individual from the next iteration of $t,$ ${x}_{r}$ refers to the place of an arbitrarily elected candidate at the present iteration, and ${x}_{b}$ and ${x}_{m}$ are optimum and averaged places $in/of$ the swarms. ${r}_{1},$ ${r}_{2}$, and ${r}_{3}$ are 3 arbitrary numbers from the Gauss distribution. $q$ implies the chance of an individual following that most 2 manners, which represents which it can also be an arbitrary number^29^. The energy of rabbits, signified as symbol $E$, declines linearly in the maximal value to 0^30–32^ in Eq. (11):

11 $$E=2{E}_{0}\left(1-\frac{\tau }{\mathrm{ max}Iter}\right)$$

where ${E}_{0}$ refers to the primary phase of energy that also fluctuates in the interval of [0, 1]. $maxIter$ stands for the maximal permitted iteration number that is set up at the start. When $|E|<$ $1$, the Harris hawk is a manner for rabbits with approaches that are explained. $\tau$ refers the current iteration.

Soft besiege. $If|E|\ge 0.5$ and $r\ge 0.5$, the Harris hawk encloses rabbits softly and scares rabbits to run to make the rabbits tired. During this approach, the Harris hawk is upgrading its places with the subsequent formula in Eq. (12):

12 $${x}_{i}\left(t+1\right)={x}_{b}-{x}_{i}\left(T\right)-E\left|\mathrm{J}\cdot {x}_{b}-{x}_{i}\left(t\right)\right|$$

where $J=2(1-{r}_{\mathrm{s}})$ signified the arbitrary jumps near the rabbit.

Hard besiege. $if|E|<0.5$, and $r\ge 0.5$, the rabbits were previously exhausted to minimal energy; afterward, the Harris hawk was carrying out hard besiege and made the surprised pounce in Eq. (13).

13 $${x}_{i}\left(T+1\right)={x}_{b}-E\left|{x}_{b}-{x}_{i}\left(t\right)\right|$$

Soft besiege with progressive rapid dives. If $|E|\ge 0.5$ and $r<0.5$, the rabbits are sufficient energy; therefore, the Harris hawks until soft besiege is performed, but in further intelligence, one in Eqs. (14)–(16):

14 $${x}_{i}\left(T+1\right)=\left\{\begin{array}{l}Y f\left(Y\right)<F\left({x}_{i}\left(t\right)\right)\\ Z f\left(Z\right)<F\left({x}_{i}\left(\mathrm{t}\right)\right)\end{array}\right.$$

15 $$Y={x}_{b}-E\left|\mathrm{J}\cdot {x}_{b}-{x}_{i}\left(t\right)\right|$$

16 $$Z=Y+S\times LF\left(D\right)$$

where $D$ implies the dimensionality of the issues, and $S$ refers to the arbitrary number. $LF(x)$ shows the Levy flight from $D$ dimensions in Eq. (17):

17 $$LF\left(x\right)=0.01\times \frac{\mu \times \sigma }{{\left|v\right|}^{\frac{1}{\beta }}}, \sigma ={\left(\frac{\Gamma \left(1+\beta \right)\times \mathrm{ sin }\left(\frac{\pi \beta }{2}\right)}{\Gamma \left(\frac{1+\beta }{2}\right)\times \beta \times 2\left(\frac{\beta -1}{2}\right)}\right)}^{\frac{1}{\beta }}$$

Hard besiege with progressive rapid dives. If $|E|<0.5$ and $r<0.5$, the rabbits were tired, and Harris hawks were performed hard besiege with intelligence. The influence of the upgrading formulas is similar to Eq. (15), but the middle parameter of $Y$ is altered, which exists to the averages ${x}_{m}$ in Eq. (18):

18 $$Y={x}_{b}-E\left|J\cdot {x}_{b}-{x}_{m}\right|$$

## Methods

**Figure 3:**
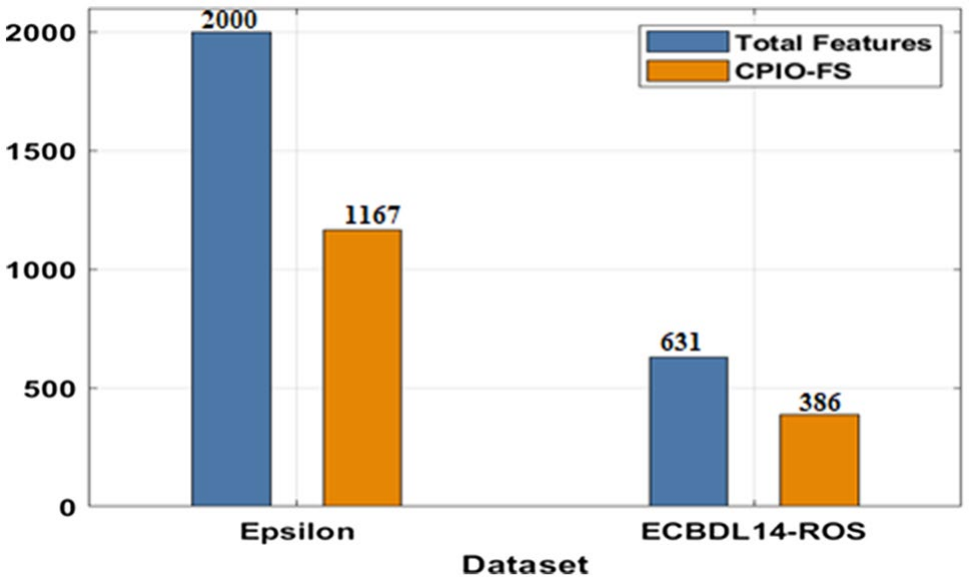
FS analysis of CPIO-FS model. The results depicted that the CPIO-FS technique has chosen an optimal set of features from the applied dataset. For instance, on the test Epsilon dataset, the CIPO-FS technique selected a set of 1167 features out of 2000 features. In addition, on the applied ECBDL14-ROS technique, the CIPO-FS technique has chosen a collection of 631 features out of 386 features.

**Figure 4:**
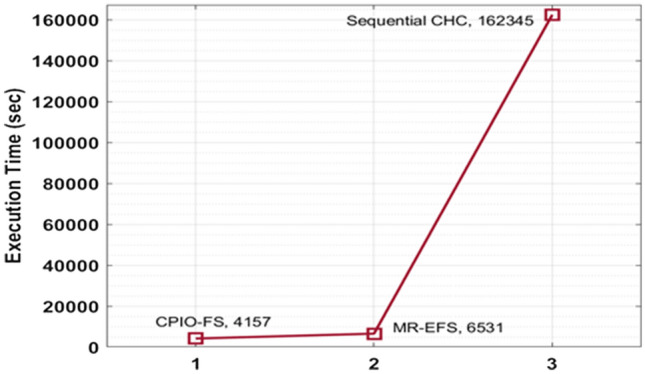
Execution time analysis of CPIO-FS model. The results showed that the CPIO-FS technique obtained an effective outcome with a minimal execution time of 4157 s, whereas the MR-EFS and sequential CHC techniques achieved higher execution times of 6531 s and 162,345 s, respectively.

**Figure 5:**
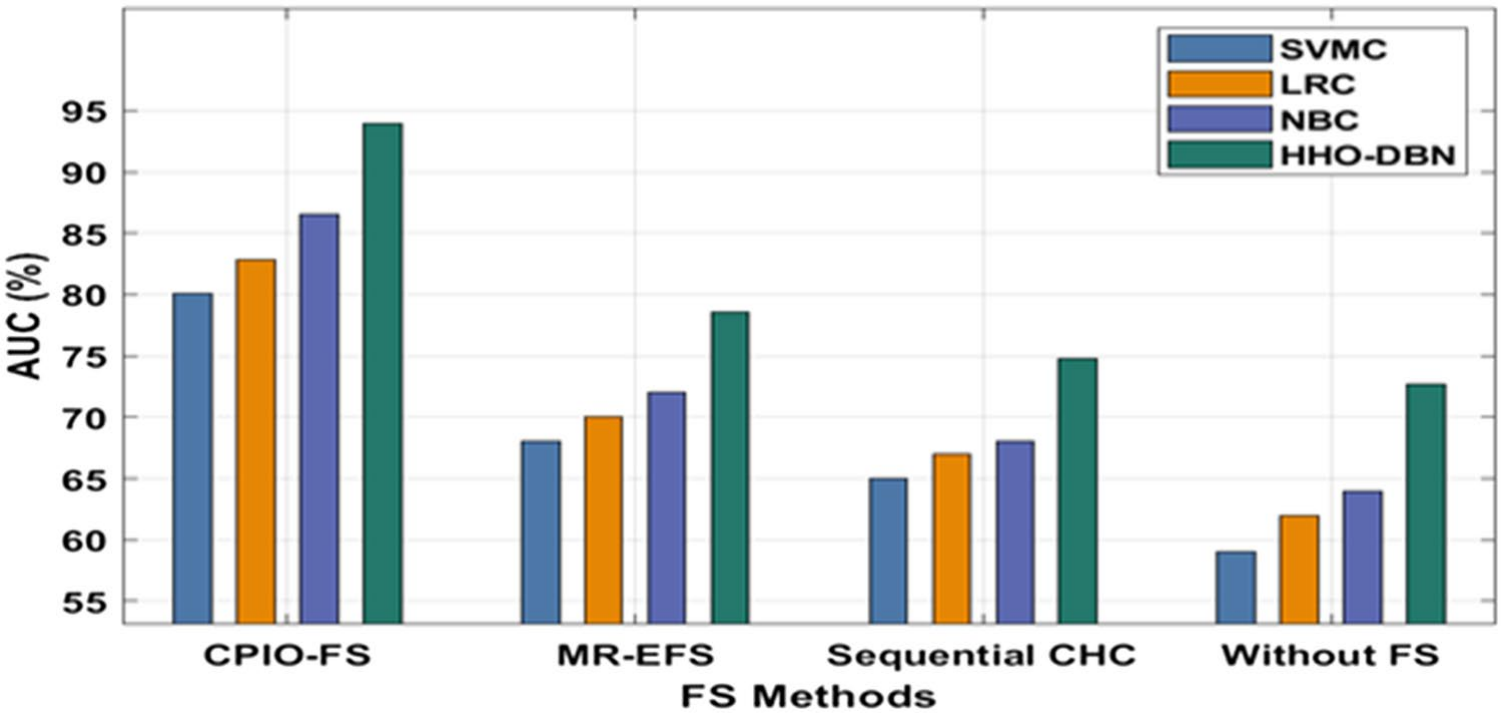
AUC analysis of HHO-DBN model under epsilon dataset. The results ensured that the CPIO-HS with the HHO-DBN model offered the maximum outcome over the other techniques. For instance, with no FS technique, the HHO-DBN model attained a higher AUC of 72.70%, whereas the SVMC, LRC, and NBC techniques attained a lower AUC of 59%, 62%, and 64%, respectively. Following the sequential CHC approach, the HHO-DBN manner reached an increased AUC of 74.80%, whereas the SVMC, LRC, and NBC methods obtained minimal AUCs of 65%, 67%, and 68%, respectively. In line with MR-EFS, the HHO-DBN method reached a superior AUC of 78.60%, whereas the SVMC, LRC, and NBC methodologies achieved a minimal AUC of 68%, 7%, and 72%, respectively. Last, with the CPIO-FS technique, the HHO-DBN methodologies reached a superior AUC of 93.90%, whereas the SVMC, LRC, and NBC approaches attained minimal AUCs of 80.10%, 82.80%, and 86.50%, respectively.

**Figure 6:**
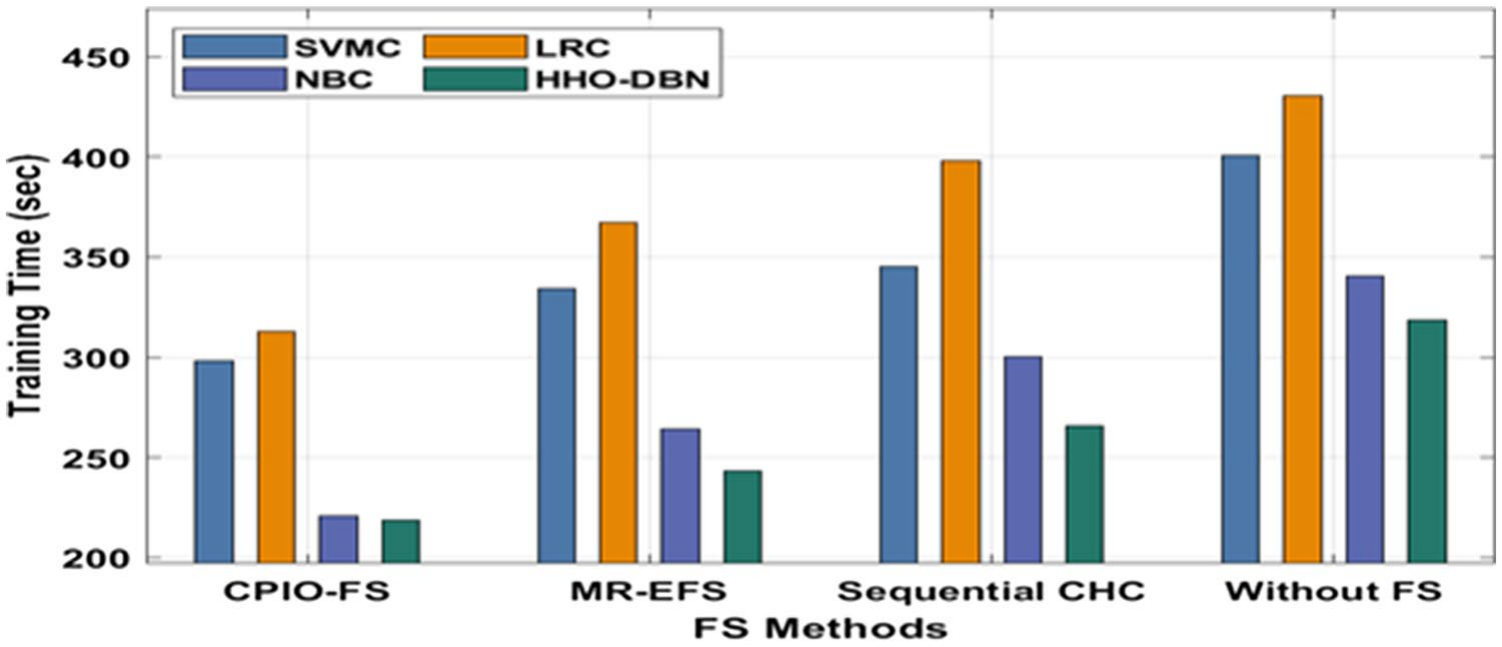
Training time analysis of the HHO-DBN model under the epsilon dataset. The results portrayed that CPIO-FS with the HHO-DBN technique resulted in the least training runtime compared to the other methods. For instance, with no FS, the HHO-DBN technique has gained a lower runtime of 318.54 s, whereas the SVMC, LRC, and NBC techniques have accomplished a higher runtime of 400.38 s, 430.48 s, and 340.42 s, respectively. Moreover, sequential CHC, the HHO-DBN approach has attained a minimal runtime of 265.98 s, whereas the SVMC, LRC, and NBC approaches have accomplished superior runtimes of 345.27 s, 398.07 s, and 300.21 s, respectively. Furthermore, MR-EFS, the HHO-DBN method has reached an increased runtime of 243.09 s, whereas the SVMC, LRC, and NBC methodologies have accomplished a higher runtime of 334.18 s, 367.29 s, and 264.26 s, respectively. Finally, CPIO-FS, the HHO-DBN method has attained a lower runtime of 218.96 s, whereas the SVMC, LRC, and NBC algorithms have accomplished superior runtimes of 298.36 s, 312.78 s, and 220.90 s, respectively.

**Figure 7:**
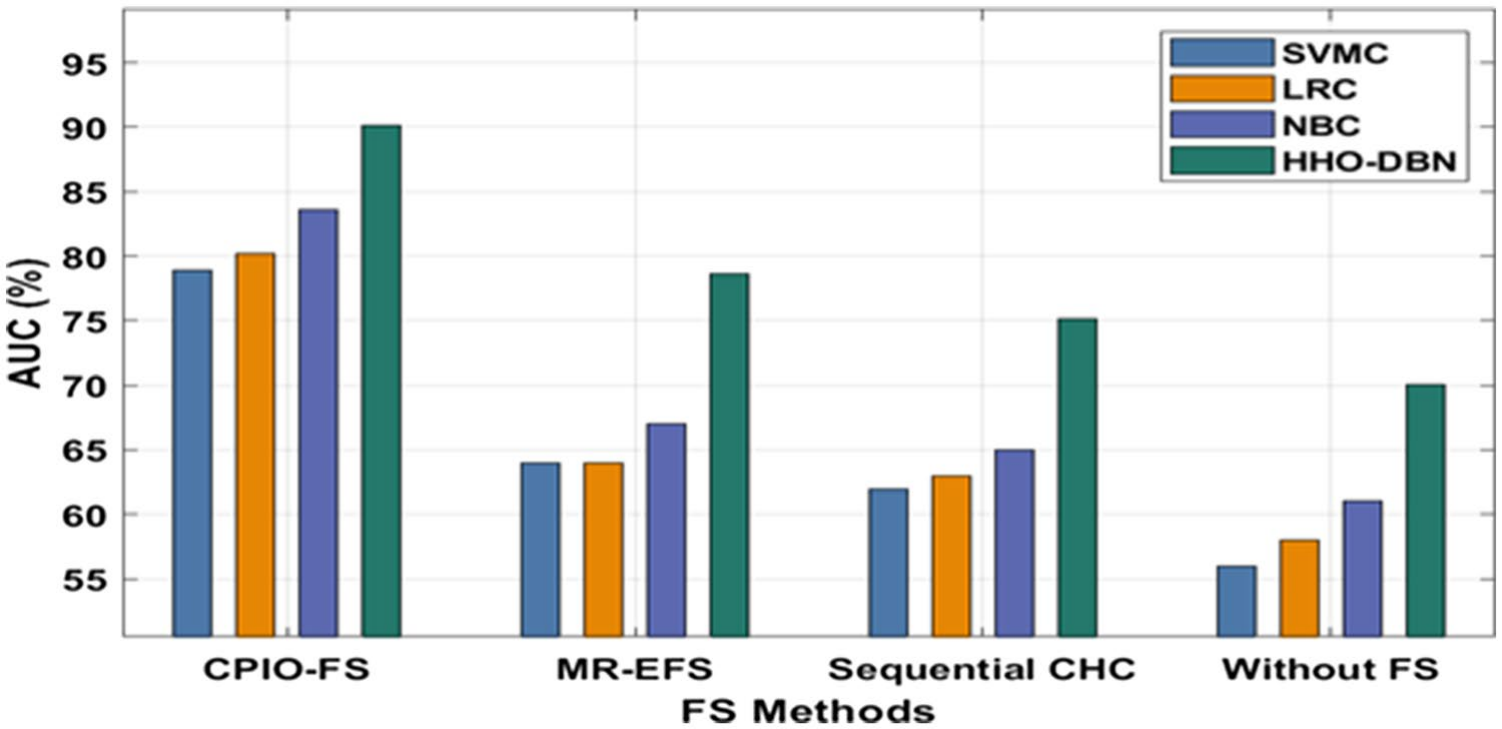
AUC analysis of HHO-DBN model under ECBDL14-ROS dataset. The outcomes demonstrated that CPIO-FS with the HHO-DBN technique resulted in the lowest training AUC compared to the other methods. For instance, with no FS, the HHO-DBN manner has reached a maximum AUC of 70.10%, whereas the SVMC, LRC, and NBC techniques have accomplished a lower AUC of 56%, 58%, and 61%, respectively. Additionally, sequential CHC and the HHO-DBN technique achieved a higher AUC of 75.14%, whereas the SVMC, LRC, and NBC techniques achieved a minimum AUC of 62%, 63%, and 65%, respectively. Likewise, MR-EFS, the HHO-DBN technique, gained an increased AUC of 78.60%, whereas the SVMC, LRC, and NBC techniques accomplished a decreased AUC of 64%, 64%, and 67%, respectively. Finally, CPIO-FS and the HHO-DBN technique achieved a higher AUC of 90.10%, whereas the SVMC, LRC, and NBC techniques achieved minimal AUCs of 78.90%, 80.15%, and 83.60%, respectively.

**Figure 8:**
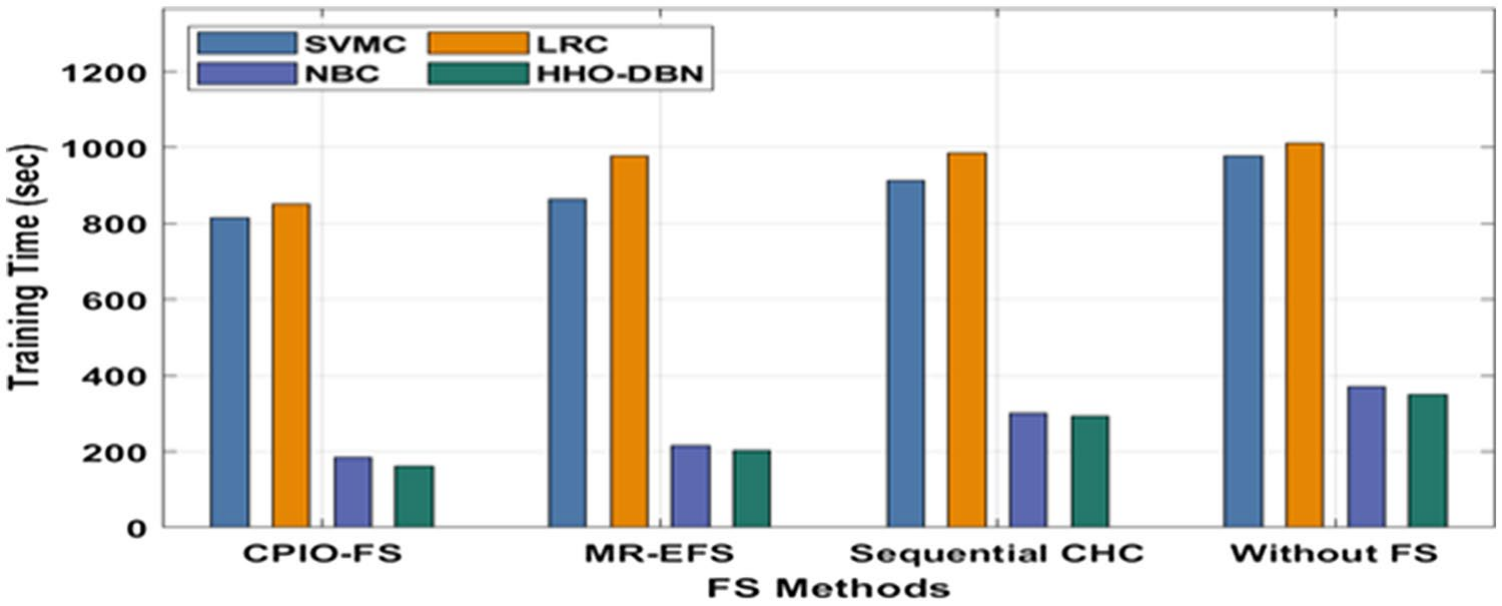
Training time analysis of the HHO-DBN model under the ECBDL14-ROS dataset. The results portrayed that CPIO-FS with the HHO-DBN technique resulted in the least training runtime compared to the other techniques. For instance, with no FS, the HHO-DBN technique has gained a minimal runtime of 350.76 s, whereas the SVMC, LRC, and NBC methods have accomplished superior runtimes of 978.37 s, 1012.47 s, and 369.98 s, respectively. In addition, sequential CHC, the HHO-DBN approach has reached a reduced runtime of 293.87 s, whereas the SVMC, LRC, and NBC algorithms have accomplished superior runtimes of 912.40 s, 986.45 s, and 300.26 s, respectively. In addition, MR-EFS, the HHO-DBN method has reached a lesser runtime of 203.76 s, whereas the SVMC, LRC, and NBC methodologies have accomplished maximal runtimes of 864.28 s, 978.38 s, and 215.09 s, respectively. Last, CPIO-FS, the HHO-DBN manner, obtained a minimal runtime of 160.90 s, whereas the SVMC, LRC, and NBC methodologies accomplished superior runtimes of 815.89 s, 850.53 s, and 184.34 s, respectively.

The evaluation of the experimental results of the presented technique takes place on two benchmark datasets, namely, Epsilon and ECBDL14-ROS. The former dataset has 400,000 samples, whereas the latter dataset has 65,003,913 samples^33^, as shown in Table 1. Figure 3 offers the FS outcome of the CIPO-FS technique. Figure 4 demonstrates the execution time analysis of the CPIO-FS technique with existing techniques with 400,000 training instances. Figure 5 investigates the AUC analysis of different classification models under different FS approaches on the epsilon dataset^34,35^. A comprehensive training runtime analysis of different classification models under different FS approaches on the epsilon dataset is provided in Fig. 6. A brief AUC analysis of different classification models under distinct FS methods on the ECBDL14-ROS dataset is provided in Fig. 7. A detailed training runtime analysis of different classification approaches under different FS approaches on the ECBDL14-ROS dataset is provided in Fig. 8.

**Table 1:** Dataset description.

Dataset	Training instances	Test instances	Features
Epsilon	400,000	100,000	2000
ECBDL14-ROS	65,003,913	2,897,917	631

## Conclusion

In this study, a new big data classification model is designed in the MapReduce environment. The proposed model derived a novel CPIO-based FS technique, which extracts a useful subset of features. In addition, the HHO-DBN model receives the chosen features as input and performs the classification process. The design of the HHO-based hyperparameter tuning process assists in enhancing the classification results to a maximum extent. To examine the superiority of the presented technique, a series of simulations were performed, and the results were inspected under various dimensions. The resultant values highlighted the supremacy of the presented technique over the recent techniques. The proposed work utilized a limited number of quality service parameters for the implementation. In the future, hybrid metaheuristic algorithms and advanced DL architectures can be designed to further improve the classification outcomes of big data with an extended number of quality of service parameters.

## Data Availability

Data are available upon reasonable request for researchers who meet the criteria for access to confidential data.
